# Estimating evolutionary distances between genomic sequences from spaced-word matches

**DOI:** 10.1186/s13015-015-0032-x

**Published:** 2015-02-11

**Authors:** Burkhard Morgenstern, Bingyao Zhu, Sebastian Horwege, Chris André Leimeister

**Affiliations:** University of Göttingen, Department of Bioinformatics, Goldschmidtstr. 1, Göttingen, 37073 Germany; Université d’Evry Val d’Essonne, Laboratoire Statistique et Génome, UMR CNRS 8071, USC INRA 23 Boulevard de France, Evry, 91037 France; University of Göttingen, Department of General Microbiology, Grisebachstr. 8, Göttingen, 37073 Germany

**Keywords:** *k*-mers, Spaced words, Alignment-free, Phylogeny, Word frequency, Distance estimation, Variance, Genome comparison

## Abstract

Alignment-free methods are increasingly used to calculate evolutionary distances between DNA and protein sequences as a basis of phylogeny reconstruction. Most of these methods, however, use heuristic distance functions that are not based on any explicit model of molecular evolution. Herein, we propose a simple estimator *d*_*N*_ of the evolutionary distance between two DNA sequences that is calculated from the number *N* of (spaced) word matches between them. We show that this distance function is more accurate than other distance measures that are used by alignment-free methods. In addition, we calculate the variance of the normalized number *N* of (spaced) word matches. We show that the variance of *N* is smaller for spaced words than for contiguous words, and that the variance is further reduced if our spaced-words approach is used with multiple patterns of ‘match positions’ and ‘don’t care positions’. Our software is available online and as downloadable source code at: http://spaced.gobics.de/.

## Background

Alignment-free methods are increasingly used for DNA and protein sequence comparison since they are much faster than traditional alignment-based approaches [1]. Applications of alignment-free approaches include *protein classification* [2-5], *read alignment* [6-8], *isoform quantification* from RNAseq reads [9], *sequence assembly* [10], *read-binning* in metagenomics [11-16] or analysis of *regulatory elements* [17-20]. Most alignment-free algorithms are based on the *word* or *k-mer composition* of the sequences under study [21]. To measure pairwise distances between genomic or protein sequences, it is common practice to apply standard metrics such as the *Euclidean* or the *Jensen-Shannon (JS)* distance [22] to the relative word frequency vectors of the sequences.

Recently, we proposed an alternative approach to alignment-free sequence comparison. Instead of considering *contiguous* subwords of the input sequences, our approach considers *spaced words, i.e.* words containing *wildcard* or *don’t care* characters at positions defined by a pre-defined *patternP*. This is similar as in the *spaced-seeds* approach that is used in database searching [23]. As in existing alignment-free methods, we compared the (relative) frequencies of these spaced words using standard distance measures [24]. In [25], we extended this approach by using whole sets $\mathcal {P} = \{P_{1},\dots,P_{m}\}$ of patterns and calculating the spaced-word frequencies with respect to *all* patterns in . In this *multiple-pattern* approach, the distance between two sequences is defined as the *average* of the distances based on the *individual* patterns $P_{i}\in {\mathcal {P}}$, see also [26]. ‘Spaced words’ have been proposed simultaneously by Onodera and Shibuya for protein classification [27] and by Ghandi *et al.* to study regulatory elements [28,29].

*Phylogeny reconstruction* is an important application of alignment-free sequence comparison. Consequently, most alignment-free methods were benchmarked by applying them to phylogeny problems [30-35]. The distance metrics used by these methods, however, are only rough measures of dissimilarity, not derived from any explicit model of molecular evolution. This may be one reason why distances calculated by alignment-free algorithms are usually not directly evaluated, but are used as input for distance-based phylogeny methods such as *Neighbour-Joining* [36]. The resulting tree topologies are then compared to trusted reference topologies. For applications to genomic sequences, we modified our distance *d*_*N*_ by taking into account that sequences can contain repeats and homologies on different strands. Obviously, this is only a very rough way of evaluating sequence-comparison methods, since the resulting tree topologies not only depend on the distance values calculated by the evaluated methods, but also on the tree-reconstruction method that is applied to them. Also, comparing topologies ignores branch lengths, so the results of these benchmark studies depend only indirectly on the distance values calculated by the alignment-free methods that are to be evaluated.

Three remarkable exceptions are the papers describing *K*_*r*_ [37], *Co-phylog* [38] and *andi* [39]. *K*_*r*_ estimates evolutionary distances based on *shortest unique substrings*, *Co-phylog* uses so-called *microalignments* defined by *spaced-word* matches and considers the *don’t care positions* to estimate distances, while *andi* uses gap-free local alignments bounded by maximal unique matches. To our knowledge, these approaches are the only alignment-free methods so far that try to estimate phylogenetic distances in a rigorous way, based on a probabilistic model of evolution. Consequently, the authors of *K*_*r*_, *Co-phylog* and *andy* compared the distance values calculated by their methods directly to reference distances. Haubold *et al.* could show that *K*_*r*_ can correctly estimate evolutionary distances between DNA sequences up to around 0.5 mutations per site [37].

In previous papers, we have shown, that our *spaced-word* approach is useful for phylogeny reconstruction. Tree topologies calculated with *Neighbour-Joining* based on *spaced-word* frequency vectors are usually superior to topologies calculated from the *contiguous* word frequency vectors that are used by traditional alignment-free methods [25]. Moreover, we could show that the ‘multiple-pattern approach’ leads to much better results than the ‘single-pattern approach’; these results were confirmed by Noé and Martin [40]. We also showed experimentally that distance values and tree topologies produced from *spaced-word* frequencies are statistically more stable than those based on *contiguous* words. In fact, the main difference between our *spaced words* and the commonly used *contiguous words* is that spaced-word matches at neighbouring positions are statistically less dependent on each other.

Since the aim of our previous papers was to compare (multiple) spaced-word frequencies to contiguous word frequencies, we applied the same distance metrics to our spaced-word frequencies that are applied by standard methods to *k*-mer frequencies, namely *Jensen-Shannon* and the *Euclidean* distance. In the present paper, we propose a new pairwise distance measure based on a probabilistic model of DNA evolution. We estimate the evolutionary distance between two nucleic-acid sequences based on the number *N* of space-word matches between them. We show that this distance measure is more accurate and works for more distantly related sequences than existing alignment-free distance measures. In addition, we calculate the *variance* of *N* for *contiguousk*-mers, as well as for *spaced words* using our single and multiple pattern approaches. We show that the variance of *N* is lower for spaced words than for contiguous words and that the variance is further reduced if *multiple* patterns are used.

This paper is an extended version of a manuscript that was first published in the proceedings of *Workshop on Algorithms in Bioinformatics (WABI) 2013* in Wroclaw, Poland [41]. We added two extensions to our WABI paper that are crucial if our method is to be applied to real-world genomic sequences. (a) While the original version of our distance function assumed that homologies are located on the same *strand* of two genomes under comparison, we modified our original distance measure to account for homologies that are on different strands. (b) The number *N* of spaced-word matches is highly sensitive to *repeats* in the compared sequences, and our previously defined distance function could grossly under-estimate phylogenetic distances in the present of repeats. We therefore propose a simple modification of this distance function that is insensitive to repeats. Finally, we added more test data sets to evaluate our method.

## Motifs and spaced words

As usual, for an alphabet *Σ* and *ℓ*∈**N**, *Σ*^*ℓ*^ denotes the set of all sequences of length *ℓ* over *Σ*. For a sequence *S*∈*Σ*^*ℓ*^ and 0<*i*≤*ℓ*, *S*[*i*] denotes the *i*-th character of *S*. A *pattern* of length *ℓ* is a word *P*∈{0,1}^*ℓ*^, *i.e.* a sequence over {0,1} of length *ℓ*. In the context of our work, a position *i* with *P*[*i*]=1 is called a *match position* while a position *i* with *P*[*i*]=0 is called a *don’t care position*. The number of all *match positions* in a patterns *P* is called the *weight* of *P*. For a pattern *P* of weight *k*, $\hat {P}_{i}$ denotes the *i*-th match position and $\hat {P} = \{\hat {P}_{1}, \dots \hat {P}_{k}\}, \hat {P}_{i} < \hat {P}_{i+1},$ denotes the set of all match positions.

A *spaced wordw* of weight *k* over an alphabet *Σ* is a pair (*P*,*w*^′^) such that *P* is a pattern of weight *k* and *w*^′^ is a word of length *k* over *Σ*. We say that a spaced word (*P*,*w*^′^) occurs at position *i* in a sequence *S* over *Σ*, if $S[i+\hat {P}_{r}-1] = w'[r-1]$ for all 1≤*r*≤*k*. For example, for

$$\Sigma = \{A,T,C,G\},\ \ P = 1101, \ \ w'= ACT, $$

we have $\hat {P} = \{1,2,4\}$, and the spaced word *w*=(*P*,*w*^′^) occurs at position 2 in sequence *S*=*CACGTCA* since

$$ S[2] S[3] S[5] = ACT = w'. $$

A pattern is called *contiguous* if it consists of match positions only, a spaced word is called contiguous if the underlying pattern is contiguous. So a ‘contiguous spaced word’ is just a ‘word’ in the usual sense.

For a pattern *P* of weight *k* and two sequences *S*_1_ and *S*_2_ over an alphabet *Σ*, we say that there is a *spaced-word match* with respect to *P* – or a *P*-match – at (*i*,*j*) if

$$ S_{1}\left[i+\hat{P}_{r}-1\right] = S_{2}\left[j+\hat{P}_{r}-1\right] $$

holds for all 1≤*r*≤*k*. For example, for sequences

$$S_{1} = ACTACAG \text{ and} S_{2}= TATAGG $$

and *P* as above, there is a *P*-match at (3,1) since one has *S*_1_[3]=*S*_2_[1],*S*_1_[4]=*S*_2_[2] and *S*_1_[6]=*S*_2_[4]. For a set ${\mathcal {P}} = \{P_{1}, \dots, P_{m}\}$ of patterns, we say that there is a -match at (*i*,*j*) if there is some $P\in {\mathcal {P}}$ such that there is a *P*-match at (*i*,*j*).

## The number *N* of spaced-word matches for a pair of sequences with respect to a set  of patterns

We consider sequences *S*_1_ and *S*_2_ as above and a fixed set $\mathcal {P} = \{P_{1},\dots,P_{m}\}$ of patterns. For simplicity, we assume that all patterns in  have the same length *ℓ* and the same weight *k*. For now, we use a simplified model of sequence evolution without insertions and deletions, with a constant mutation rate and with different sequence positions independent of each other. Moreover, we assume that we have the same substitution rates for all substitutions *a*→*b*,*a*≠*b*. We therefore consider two sequences *S*_1_ and *S*_2_ of the same length *L* with match probabilities

$$P(S_{1}[i] = S_{2}[j]) = \left\{ \begin{array}{ll} p & \text{for } i=j \\ q & \text{for } i\not=j \\ \end{array} \right. $$

If *q*_*a*_ is the relative frequency of a single character $a\in {\mathcal {A}}$, $q = \sum _{a\in {\mathcal {A}}}{q_{a}^{2}}$ is the background match probability, and *p*≥*q* is the match probability for a pair of ‘homologous’ positions.

For a pattern *P*, let *N*(*S*_1_,*S*_2_,*P*) be the number of pairs of positions (*i*,*j*) where there is a *P*-match between *S*_1_ and *S*_2_. We then define

$$ N = N(S_{1},S_{2},\mathcal{P}) = \sum_{P\in\mathcal{P}} N(S_{1},S_{2},P) $$

to be the sum of all *P*-matches for patterns $P\in \mathcal {P}$. Note that for two sequences, there can be *P*-matches for different patterns *P* at the same pair of positions (*i*,*j*). In the definition of *N*, we count not only the positions (*i*,*j*) where there is some *P*-match, but we count *allP*-matches with respect to all patterns in .

*N* can be seen as the *inner product* of $m\cdot |{\mathcal {A}}|^{k}$-dimensional count vectors for spaced words with respect to the set of patterns . In the special case where  consists of a *single contiguous* pattern, *i.e.* for *k*=*ℓ* and *m*=1, *N* is also called the *D*_2_ score [42]. The statistical behaviour of the *D*_2_ score has been studied under the *null model* where *S*_1_ and *S*_2_ are *unrelated* [18,43]. In contrast to these studies, we want to investigate the number *N* of spaced-word matches for *evolutionarily related* sequence pairs under a model as specified above. To this end, we define $X_{i,j}^{P}$ to be the Bernoulli random variable that is 1 if there is a *P*-match between *S*_1_ and *S*_2_ at (*i*,*j*), $P\in \mathcal {P}$, and 0 otherwise, so *N* can be written as

$$ N= \sum_{\substack{P\in\mathcal{P} \\ i,j }} X_{i,j}^{P} $$

If we want to calculate the expectation value and variance of *N*, we have to distinguish between ‘homologous’ spaced-word matches, that is matches that are due do ‘common ancestry’ and ‘background matches’ due to chance. In our model where we do not consider insertions and deletions, a *P*-match at (*i*,*j*) is ‘homologous’ if and only if *i*=*j* holds. So in this special case, we can define

$$ \mathcal{X}_{Hom} = \left\{ X_{i,i}^{P} | 1 \le i \le L-\ell+1, P\in\mathcal{P} \right\}, $$

$$ \mathcal{X}_{BG} = \left\{ X_{i,j}^{P} | 1 \le i,j \le L-\ell+1, i\not= j, P \in \mathcal{P} \right\}. $$

Note that if sequences do not contain insertions and deletions, *every* spaced-word match is either entirely a *homologous* match or entirely a *background* match. If indels are considered, a spaced-word match may involve both, homologous and background regions, and the above definitions need to be adapted. The set  of all random variables $X_{i,j}^{P}$ can be written as $ \mathcal {X} = \mathcal {X}_{\textit {Hom}} \cup \mathcal {X}_{\textit {BG}}$, the total sum *N* of spaced-word matches with respect to the set  of patterns is

$$ N = \sum_{X\in\mathcal{X}} X $$

and the expected number of spaced-word matches is

$$E(N) = \sum_{X\in \mathcal{X}_{Hom}} E(X) + \sum_{X\in \mathcal{X}_{BG}} E(X),$$

where the expectation value of a single random variable $X\in \mathcal {X}$ is

(1) $$ E(X) = \left\{ \begin{array}{ll} p^{k} & \text{if}\ \ X \in \mathcal{X}_{Hom} \\ q^{k} & \text{if}\ \ X \in \mathcal{X}_{BG} \end{array} \right.  $$

There are *L*−*ℓ*+1 positions (*i*,*i*) and (*L*−*ℓ*)·(*L*−*ℓ*+1) positions (*i*,*j*),*i*≠*j* where spaced-word matches can occur, so we obtain

(2) $$  E(N) = m \cdot \left[ (L-\ell+1) \cdot p^{k} + (L-\ell)\cdot (L-\ell+1) \cdot q^{k} \right]  $$

## Estimating evolutionary distances from the number *N* of spaced-word matches

If the *weight* of the patterns – *i.e.* the number of match positions – in the *spaced-words* approach is sufficiently large, random space-word matches can be ignored. In this case, the *Jensen-Shannon* distance between two DNA sequences approximates the number of (spaced) words that occur in one of the compared sequences, but not in the other one. Thus, if two sequences of length *L* are compared and *N* is the number of (spaced) words that two sequences have in common, their *Jenson-Shannon* distance can be approximated by *L*−*N*. Accordingly, the *Euclidean* distances between two sequences can be approximated by the square root of this value if the distance is small and *k* is large enough. For *small* evolutionary distances, the *Jensen-Shannon* distance grows therefore roughly linearly with the distance between two sequences, and this explains why it is possible to produce reasonable phylogenies based on this metric. It is clear, however, that the *Jensen-Shannon* distance is far from linear to the real distance for larger distances. We therefore propose an alternative estimator of the evolutionary distance between two sequences in terms of the number *N* of *spaced-word* matches between them.

Again, we first consider sequences without insertions and deletions. From the expected number *E*(*N*) of spaced words shared by sequences *S*_1_ and *S*_2_ with respect to a set of patterns  as given in equation (2), we obtain

(3) $$  \hat{p} = \sqrt[k]{\frac{N}{m\cdot (L-\ell+1)}- (L-\ell) \cdot q^{k}}  $$

as an estimator for the match probability *p* for sequences without indels, and with Jukes-Cantor [44] we obtain

(4) $$   d_{N}= -\frac{3}{4} \cdot \ln \left[ \frac{4}{3}\cdot \sqrt[k]{\frac{N}{m\cdot(L-\ell+1)}- (L-\ell) \cdot q^{k}} - \frac{1}{3} \right]  $$

as an estimator for the distance *d* between the sequences *S*_1_ and *S*_2_. Note that for a model without insertions and deletions, it is, of course, not necessary to estimate the mismatch probability *p* from the number *N* of spaced-word matches. In this case, one could simply *count* the mismatches between two sequences and use their relative frequency as an estimator for *p*. The reason why we want to estimate *p* based on the number of spaced-word matches is that this estimate can be easily adapted to a model with insertions and deletions.

## Local homologies, homologies on different strands and repeats

Next, we consider the case where *S*_1_ and *S*_2_ may have different lengths and share one region of *local* homology. Again, we assume again that there are no insertions and deletions within this homologous region. Let *L*_*Hom*_ be the length of the local homology; we assume that *L*_*Hom*_ is known. Equation (2) for the expected number *N* of spaced-word matches between two sequences *S*_1_ and *S*_2_ can be easily generalized to the case of local homology. If *L*_1_ and *L*_2_ are the lengths of *S*_1_ and *S*_2_, respectively, we define

$$ L^{*} = (L_{1}-\ell+1)\cdot(L_{2}-\ell+1) - L_{Hom} $$

to be the (approximate) number of positions (*i*,*j*) where a *background match* can occur (*L*^∗^ is only an approximation since we ignore spaced-word matches that involve both, homologous and background regions of the sequences). Then, we can the estimate the expected number of spaced-word matches as

$$  E(N) \approx m \cdot \left[ \left(L_{Hom}-\ell+1 \right) \cdot p^{k} + L^{*} \cdot q^{k} \right]   $$

and we obtain

(5) $$  d_{loc} = -\frac{3}{4} \cdot \ln \left[ \frac{4}{3} \cdot \sqrt[k]{\frac{N/m - L^{*} \cdot q^{k}}{L_{Hom}-\ell+1 }} - \frac{1}{3} \right]  $$

as an estimator for the distance between *S*_1_ and *S*_2_. It is straight-forward, though somewhat cumbersome, to extend this estimator to the case where the homologous region contains insertions and deletions.

Note that, if local homologies between input sequences are known to the user, the best thing would be to remove the non-homologous regions of the sequences and to use the distance *d*_*N*_ defined by equation (4) to the remaining homologous regions (which are then ‘globally’ related to each other). Nevertheless, the distance *d*_*loc*_ might be useful in situations where the *extent* of homology between genomic sequences can be estimated, even though the precise location and boundaries of these homologies are unknown.

So far, we considered the case where homologies between two genomic sequences *S*_1_ and *S*_2_ are located on the same respective strand. For realistic applications, we have to take into account that homologies can occur on both strands of the DNA double helix. More importantly, we have to consider the case where a region of homology is located on one strand of *S*_1_, but on the reverse strand on *S*_2_. Let *L*_1_ and *L*_2_ be the lengths of *S*_1_ and *S*_2_, respectively, with *L*_1_≤*L*_2_. For simplicity, we assume that the entire sequence *S*_1_ is homologous to a contiguous segment of *S*_2_ and we ignore insertions and deletions. We now assume, however, that some segments of *S*_1_ may align to their homologous counterpart in *S*_2_ while other segments of *S*_2_ may align to the reverse complement of their counterparts in *S*_2_. The more general situation involving local homology and indels can be accounted for as discussed above.

The simplest way to capture homologies between *S*_1_ and *S*_2_ regardless of their orientation is to concatenate one of the sequences, say *S*_2_ with its *reverse complement* and to compare *S*_1_ to this concatenated sequence. So in this case, we would consider all spaced-word matches between *S*_1_ and $\tilde {S_{2}}$ where $\tilde {S_{2}}$ is the concatenation of *S*_2_ and its reverse complement. To estimate the expected number of spaced-word matches in this situation, we can homologous spaced-word matches can be locate and ≈2·(*L*_1_−*ℓ*+1)·(*L*_2_−*ℓ*) positions where background matches can occur. By adapting Formulae (2) to (4) accordingly and ignoring fringe effects, we obtain

(6) $$ \begin{aligned} E(N) \approx m \cdot& \left[ \left(L_{1}-\ell+1 \right) \cdot p^{k} + 2\cdot \left(L_{1}-\ell+1 \right)\right. \\ &\quad\left.\times \left(L_{2}-\ell\right) \cdot q^{k} \right]  \end{aligned}  $$

(7) $$  \hat{p} = \sqrt[k]{\frac{N}{m\cdot (L_{1}-\ell+1)} - 2 \cdot (L_{2}-\ell) \cdot q^{k}}  $$

as an estimator for the match probability *p* for sequences without indels, and with Jukes-Cantor [44] we obtain

(8) $$  {\fontsize{8.8}{6} \begin{aligned} d_{RC} = -\frac{3}{4} \cdot \ln \left[ \frac{4}{3} \cdot \sqrt[k]{\frac{N}{m\cdot\left(L_{1}-\ell+1\right)} - 2 \cdot \left(L_{2}-\ell\right) \cdot q^{k}} - \frac{1}{3} \right] \end{aligned}}  $$

as an estimator for the distance *d* between the sequences *S*_1_ and *S*_2_ if homologies on the *reverse complement* are taken into account.

Finally, we consider the case where sequences contain *repeats*. A direct application of the distance functions discussed so far would be highly sensitive to repeats in the input sequences, since repeats can drastically increase the number *N* of (spaced) word matches. This can even lead to negative distance values if the number *N* of  matches between two sequences with repeats exceeds the expected number of  matches between a non-repetitive sequence of the same length to itself. A simple but efficient way of dealing with repeats is to use *binary* variables *N*^*bin*^(*S*_1_,*S*_2_,*P*) that are one if there are one or several *P* matches between sequences *S*_1_ and *S*_2_, and zero if there is no such match. Instead of using the number *N* of  matches for a set  of patterns, we then consider

$$ N^{bin} = N^{bin}\left(S_{1},S_{2},\mathcal{P}\right) = \sum_{P\in\mathcal{P}} N^{bin}\left(S_{1},S_{2},P\right) $$

and distances $d_{N}^{bin}$, $d_{\textit {loc}}^{bin}$ and $d_{\textit {RC}}^{bin}$, respectively, can be defined as in equations (4), (5) and (8), but with *N* replaced by *N*^*bin*^.

## The variance of *N*

Our new distance measure and other word-based distance measures depend on the number *N* of (spaced) word matches between sequences. To study the stability of these measures, we want to calculate the *variance* of *N*. To do so, we adapt results on the occurrence of words in a sequence as outlined in [45]. Since *N* can be written as the sum of all random variables $X_{i,j}^{P}$, we need to calculate the *covariances* of these random variables. To simplify this, we make a further assumption on our sequence model: we assume that the four nucleotides occur with the same probability 0.25. In this case, the covariance of two random variables $X_{i,j}^{P}$ and $X_{i',j'}^{P'}$ can be non-zero only if *i*^′^−*i*=*j*^′^−*j* holds (note that this is not true if nucleotides have different probabilities to occur). In particular, for random variables $X\in \mathcal {X}_{\textit {Hom}}$ and $X'\in \mathcal {X}_{\textit {BG}}$, their covariance is zero. Thus, we only need to consider covariances of pairs of random variables $X_{i,j}^{P}$ and $X_{i+s,j+s}^{P'}$.

For patterns *P*,*P*^′^ and *s*∈**N** we define *n*(*P*,*P*^′^,*s*) to be the number of integers that are match positions of *P* or match positions of *P*^′^ shifted by *s* positions to the right (or both). Formally, if

$$ \hat{P}_{s} = \left\{\hat{P}_{1} + s,\dots, \hat{P}_{k} + s\right\} $$

denotes the set of match positions of a pattern *P* shifted by *s* positions to the right, we define

$$ n(P,P',s) = |\hat{P} \cup \hat{P'}_{s}| = |\hat{P}| + |\hat{P'}_{s}| - |\hat{P} \cap \hat{P'}_{s}| $$

For example, for *P*=101011,*P*^′^=111001 and *s*=2, there are 6 positions that are match positions of *P* or of *P*^′^ shifted by 2 positions to the right, namely positions 1, 3, 4, 5, 6, 8:

$$\begin{array}{cccccccccc} P: & & 1 & 0 & 1 & 0 & 1 & 1 & & \\ P': & & & & 1 & 1 & 1 & 0 & 0 & 1\\ \end{array} $$

so one has *n*(*P*,*P*^′^,*s*)=6. In particular, one has *n*(*P*,*P*,0)=*k* for all patterns *P* of weight *k*, and

$$ n(P,P,s) = k + \max\{s,k\} $$

for all *contiguous* patterns *P* of weight (or length) *k*. With this notation, we can write

(9) $$\begin{array}{@{}rcl@{}}  E\left(X_{i,j}^{P} \cdot X_{i+s,j+s}^{P'} \right) & = &\left\{ \begin{array}{ll} p^{n(P,P',s)} & \text{if}\ \ i = j\\ q^{n(P,P',s)} & \text{else} \end{array} \right. \end{array} $$

for all $X_{i,j}^{P}, X_{i+s,j+s}^{P'}$

To calculate the covariance of two random variables from , we distinguish again between homologous and random matches. We first consider ’homologous’ pairs $X_{i,i}^{P}, X_{i+s,i+s}^{P'} \in \mathcal {X}_{\textit {Hom}}$. Here, we obtain with (9)

(10) $$  \begin{aligned} Cov \left(X_{i,i}^{P}, X_{i+s,i+s}^{P'}\right) &= p^{n(P,P',s)} - p^{2k} \end{aligned}  $$

Similarly, for a pair of ‘background’ variables $X_{i,j}^{P},$$ X_{i+s,j+s}^{P'} \in \mathcal {X}_{\textit {BG}}$, one obtains

(11) $$  \begin{aligned} Cov \left(X_{i,j}^{P}, X_{i+s,j+s}^{P'}\right) &= q^{n(P,P',s)} - q^{2k}. \end{aligned}  $$

Since ‘homologous’ and ‘background’ variables are uncorrelated, the variance of *N* can be written as

$$\begin{aligned} Var(N) =&\; Var \left(\sum_{X\in\mathcal{X}} X\right) = Var \left(\sum_{X\in \mathcal{X}_{Hom}}\right)\\ &+ Var \left(\sum_{X\in \mathcal{X}_{BG}}\right) \end{aligned} $$

We express the variance of these sums of random variable as the sum of all of their covariances, so for the ’homologous’ random variables we can write

$$ Var \left(\sum_{X\in \mathcal{X}_{Hom}}X\right) = \sum_{P,P'\in\mathcal{P}} \sum_{i,i'=1}^{L-l+1} Cov \left(X_{i,i}^{P}, X_{i',i'}^{P'} \right) $$

Since the covariance for non-correlated random variables vanishes, we can ignore the covariances of all pairs $\left (X_{i,i}^{P}, X_{i',i'}^{P'}\right)$ with |*i*−*i*^′^|≥*l* so, ignoring side effects, we can write the above sum as

$${\fontsize{9.3}{6}\begin{aligned} Var \left(\sum_{X\in \mathcal{X}_{Hom}} X\right) & \approx &\sum_{i=1}^{L-\ell+1} \sum_{P,P'\in\mathcal{P}} \sum_{s= -\ell + 1}^{\ell-1} Cov \left(X_{i,i}^{P}, X_{i+s,i+s}^{P'} \right) \end{aligned}} $$

and since the above covariances depend only on *s* but not on *i*, we can use (9) and (11) and obtain

$$\begin{aligned} Var &\left(\sum_{X\in \mathcal{X}_{Hom}} X\right) \approx (L-\ell+1)\\ &\times\sum_{P,P'\in\mathcal{P}} \sum_{s=-\ell+1}^{\ell-1} \left(p^{n(P,P',s)} - p^{2k}\right) \end{aligned} $$

and similarly

$$\begin{aligned} Var &\left(\sum_{X\in \mathcal{X}_{BG}}X\right) \approx (L-\ell+1) \cdot (L-\ell)\\ &\times\sum_{P,P'\in\mathcal{P}}\sum_{s=-\ell+1}^{\ell-1} \left(q^{n(P,P',s)} - q^{2k}\right) \end{aligned} $$

Together, we get

(12) $$  {\begin{aligned} Var(N) \approx & \;(L-\ell+1) \cdot \sum_{P,P'\in\mathcal{P}} \sum_{s=-\ell+1}^{\ell-1} \left(p^{n(P,P',s)} - p^{2k} \right)\\ & +\ \ (L-\ell+1) \cdot (L-\ell) \\ &\times\sum_{P,P'\in\mathcal{P}} \sum_{s=-\ell+1}^{\ell-1} \left(q^{n(P,P',s)} - q^{2k}\right) \end{aligned}}  $$

## Test results

### Simulated DNA sequences

To evaluate the distance function *d*_*N*_ defined by equation (4), we simulated pairs of DNA sequences with an (average) length of 100,000 and with an average of *d* substitutions per sequence position. More precisely, we generated sequence pairs by generating a first sequence using a Bernoulli model with probability 0.25 for each nucleotide. A second sequence was then generated from the first sequence by substituting nucleotides with a probability corresponding to the substitution frequency *d*, as calculated with Jukes-Cantor. We varied *d* between 0 and 1 and compared the distances estimated by our distance measure and by various other alignment-free programs to the ‘real’ distance *d*. We performed these experiments for sequence pairs without insertions and deletions and for sequence pairs where we included insertions and deletions with a probability of 1*%* at every position. The length of indels was randomly chosen between 1 and 50 with uniform probability.

**Figure 1:**
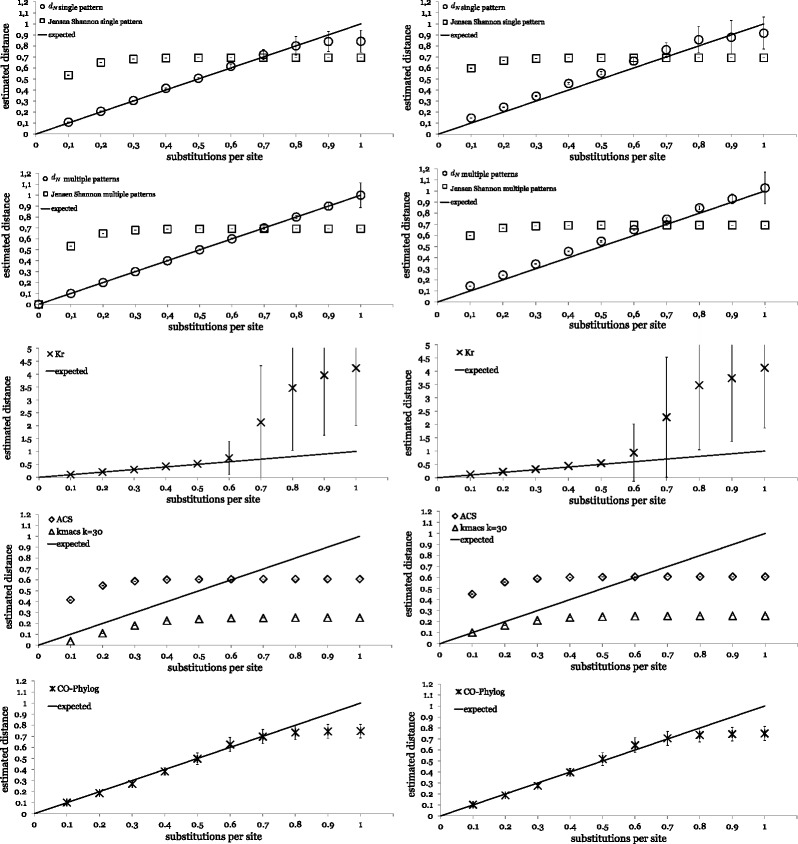
**Distances calculated by different alignment-free methods.** Distances were calculated for pairs of simulated DNA sequences and plotted against their ‘real’ distances *d* measured in substitutions per site. Plots on the left-hand side are for sequence pairs without insertions and deletions, on the right-hand side the corresponding results are shown for sequences with an indel probability of 1*%* for each site and an average indel length of 25. From top to bottom, the applied methods were: 1. spaced words with the *single-pattern* approach and the *Jensen-Shannon* distance (squares) and the distance *d*_*N*_ defined in equation (4) in this paper (circles), 2. the *multiple-pattern* version of *Spaced Words* using sets  of *m*=100 patterns with the same distance functions, 3. distances calculated with *K*_*r*_ [37], 4. with *kmacs* [47] and *ACS* [30] and 5. with *Co-phylog* [38].

Figure 1 shows the results of these experiments. Our new distance measure *d*_*N*_ applied to spaced-word frequencies is well in accordance with the real distances *d* for values of *d*≤0.8 on sequence pairs without insertions and deletions if the *single-pattern* version of our program is used. For the *multiple-pattern* version, our distance function estimates the real distances correctly for all values of *d*≤1. If indels are added as specified above, our distance functions slightly overestimates the real distance *d*. By contrast, the *Jensen-Shannon* distance applied to the same spaced-word frequencies increased non-linearly with *d* and flattened for values of around *d*≥0.4.

As mentioned, *K*_*r*_ [46] estimates evolutionary distances on the basis of a probabilistic model of evolution. In our study, *K*_*r*_ correctly estimated the true distance *d* for values of around *d*≤0.6, this precisely corresponds to the results reported by the authors of the program. For larger distances, *K*_*r*_ grossly overestimates the distance *d*, though, and the variance strongly increases. The distances estimated by *Co-phylog* [38] nearly coincide with the substitution rate *d* for values *d*≤0.7, then the curve flattens. Moreover, it appears that the distances calculated with *Co-phylog* are not much affected by indels. The distance values calculated by the program *k mismatch average common substring (kmacs)* that we previously developed [47] are roughly linear to the real distances *d* for values of up to around *d*=0.3. From around *d*=0.5 on, the curve becomes flat. With *k*=30 mismatches, the performance of *kmacs* was better than with *k*=0, in which case *kmacs* corresponds to the *Average Common Substring (ACS)* approach [30].

### Real-world genomes

Next, we applied various distance measures to a set of 27 mitochondrial genomes from different primates that were previously used by [46] as a benchmark data set for alignment-free approaches. We used our *multiple spaced-words* approach with the parameters that we used in [25], that is with a pattern weight (number of match positions) of *k*=9 and with pattern lengths *ℓ* between 9 and 39, *i.e.* with up to 30 *don’t-care* positions in the patterns. For each value of *ℓ*, we randomly generated sets  of *m*=100 patterns. For this data set, we used the distance *d*_*RC*_ defined in equation (8) that takes the reverse complement of the input sequences into account. (We did not use the ‘binary’ version $d_{\textit {RC}}^{bin}$ of our distance function, since these sequences do not contain major repeats). In addition, we used the competing approaches *FFP* [32], CVTree [48], *K*_*r*_ [37], *kmacs* [47], *ACS* [30] and Co-phylog [38]. For some of these methods, program parameters need to be defined, *e.g.* a predefined word length or the number of allowed mismatches. For these methods we tested various parameters and used the best performing values.

**Figure 2:**
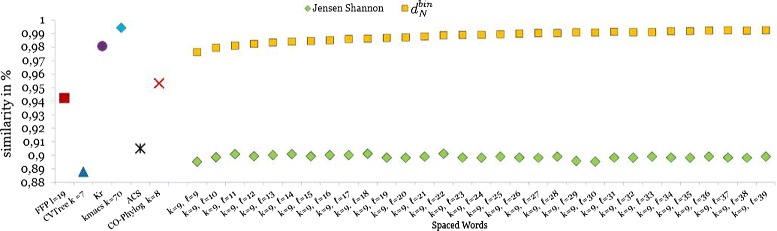
**Comparison of distance matrices for primate mitochondrial genomes.** We applied various alignment-free methods to a set of 27 mitochondrial genomes from different primates and compared the resulting distance matrices to a trusted *reference* distance matrix using the *Mantel test*. The similarity between the calculated matrices and the reference matrix is plotted. We applied our *Spaced-Words* approach using sets  of 100 randomly calculated patterns with weight *k*=9 and length *ℓ* between 9 and 39, *i.e.* with 9 *match positions* and up to 30 *don’t care* positions. Yellow squares are the results for the ‘binary’ version of new distance measure $d_{N}^{bin}$. We did not use the reverse-complement option on these data, since genes in the compared genomes are known to be on the same strand. Green diamonds are the results for the *Jensen-Shannon* distance applied to the same spaced-word frequency vectors as explained in [25]. In addition, distances calculated by six other alignment-free methods were evaluated.

With each method, we calculated a distance matrix for the input sequences, and we compared this matrix to a *reference* distance matrix that we calculated with the program *Dnadist* from the *PHYLIP* package [49] based on a reference multiple alignment. For comparison with the reference matrix, we used a software program based on the *Mantel* test [50] that was also used by Didier *et al.* [51]. Figure 2 shows the results of this comparison. As can be seen, our new distance measure *d*_*RC*_, applied to multiple spaced-word frequencies, produced distance matrices close to the reference matrix and outperformed the *Jenson-Shannon* distance for all pattern lengths *ℓ* that we tested. The distance function *d*_*RC*_ also outperformed some of the existing alignment-free methods, with the exception of *K*_*r*_ and *kmacs*.

**Figure 3:**
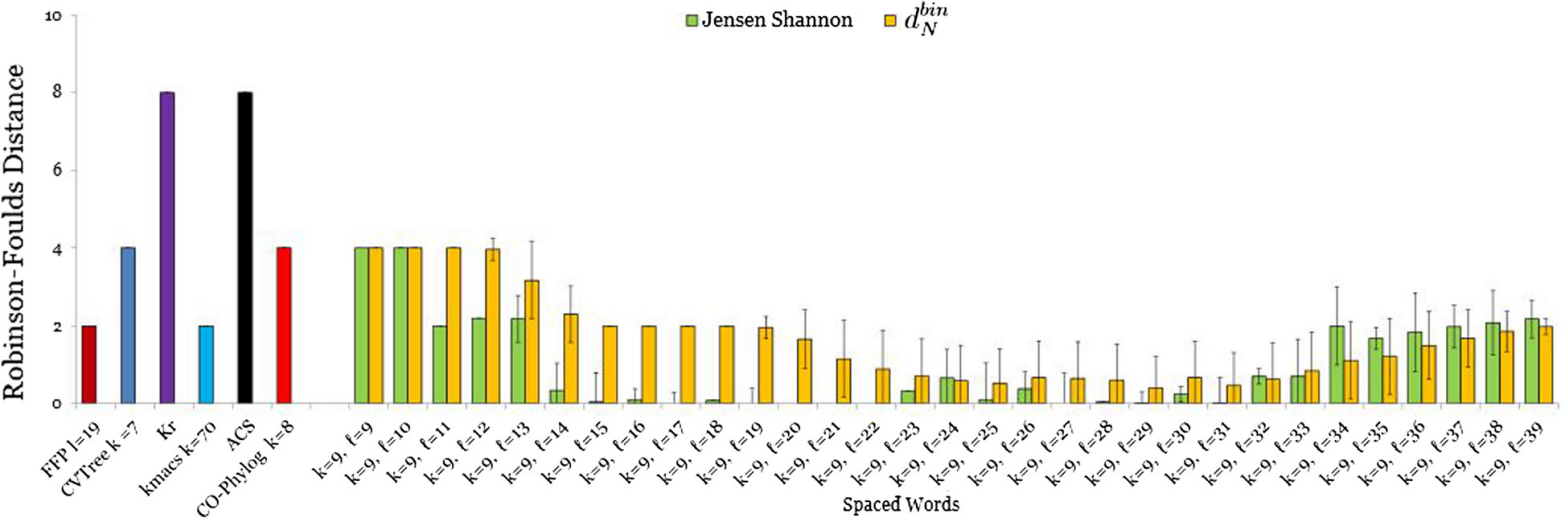
**RF distances for primate mitochondrial genomes.** Performance of various alignment-free methods on the same set of 27 primate mitochondrial genomes as in Figure 2. *Neighbour-Joining* was applied to the calculated distance matrices, the resulting tree topologies were compared with the Robinson-Foulds metric. Parameters for *Spaced Words* and colour coding as in Figure 2.

In addition to this direct distance comparison we performed an indirect evaluation by phylogeny analysis. To do so, we applied *Neighbor-Joining* [36] to the distance matrices and compared the resulting trees to the corresponding reference tree, using the *Robinson-Foulds (RF)* metric [52]. The results for the mitochondrial genomes are shown in Figure 3. The outcome of this evaluation is partially in contradiction to the results of the direct comparison of the distances. Figure 2 shows that the distance matrices produced by *Spaced-Words* with the *Jensen-Shannon divergence* are worse than the distance matrices produced by most other methods, if these matrices are directly compared to the reference distance matrix. However, *Spaced Words* with *Jensen-Shannon* led to better tree topologies than most other methods in our study, as shown in Figure 3. A similar contradictory result is observed for *K*_*r*_. While the distance matrix produced by *K*_*r*_ is similar to the reference matrix, the tree topology produced with these distances is further away from the reference topology than the trees computed by the other alignment-free approaches in our study.

To evaluate our new method for larger sequences we used two prokaryotic data sets. The first data set consists of 26 *E. coli* and *Shigella* genomes, which are very closely related and the second data set consist of 32 *Roseobacter* genomes, which are far more divergent. For these sequences, we used our ‘repeat-aware’ distance function $d_{\textit {RC}}^{bin}$. As for the primate mitochondrial genomes, we calculated distance matrices using the same alignment-free methods, constructed trees with *Neighbor-Joining* and compared the resulting tree topologies to a benchmark tree using the *RF* metric. For the *E.coli/Shigella* genomes we used the tree proposed by [53] as reference which is based on concatenated alignments of the 2034 core genes. For the *Roseobacter* genomes we used the tree by [54] as reference. This benchmark tree was constructed based on alignments of 70 universal single-copy genes.

**Figure 4:**
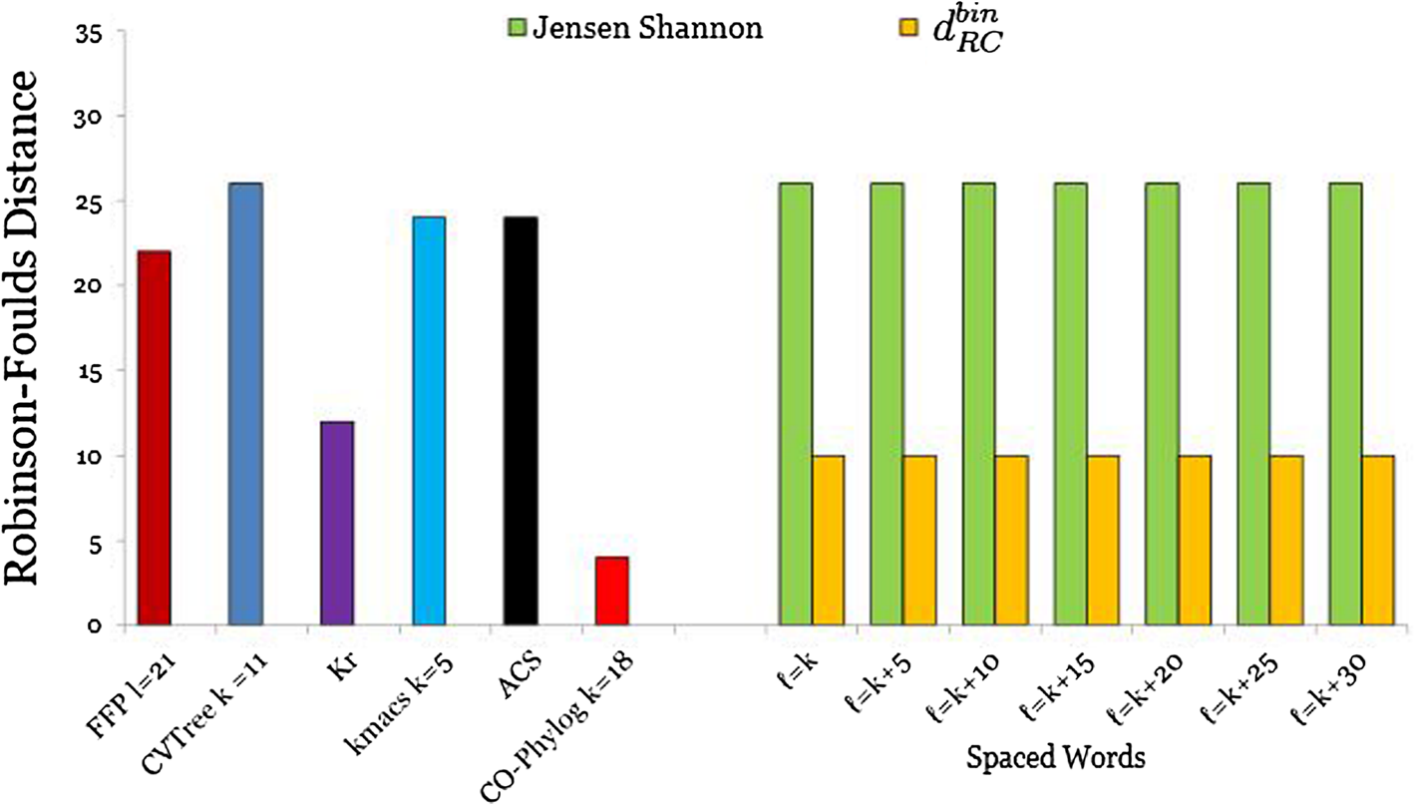
**RF distances for E.coli/Shigella genomes.** Performance of various alignment-free methods on a set of 26 E.coli/Shigella genomes. Robinson-Foulds distances to the reference tree are shown. For *Spaced Words*, we used a weight of *k*=17 and applied the ‘binary’ distance function $d_{\textit {RC}}^{bin}$. Colour coding as in Figure 2.

The results for the *E.coli/Shigella* are shown in Figure 4. The best result was achieved by *Co-phylog* with a *RF* distance of only 4, followed by our new distance *d*_*RC*_ with a *RF* distance of 10, which is a huge improvement compared to the previously described version of *Spaced Words* where we used the *Jensen-Shannon divergence*. *K*_*r*_ performed slightly worse than our new estimator with a *RF* distance of 12. The other alignment-free methods performed relatively poorly. For the *Spaced Words* approach we performed 25 runs with *m*=100 patterns that were randomly generated. For this data set, all sets of patterns led to the same tree topology. Additionally the results are also not influenced by the number of *don’t-care* positions, which can be explained by the very small number of substitutions between these genomes.

**Figure 5:**
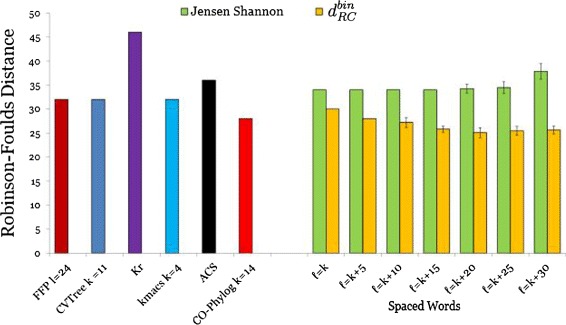
**RF distances for Roseobacter genomes.** Performance of various alignment-free methods on a set of 32 Roseobacter genomes. Robinson-Foulds distances to the reference tree are shown. *Spaced Words* was used with parameters as in Figure 4, colour coding is as in Figure 2.

For the *Roseobacter* genomes we used the same evaluation procedure as for the *E. coli/Shigella* genomes. Here, our new evolutionary distance *d*_*RC*_ outperformed the other alignment-free methods, if *don’t-care* positions are incorporated in the patterns, and the performance increased with the number of don’t-care positions, as shown in Figure 5. (Without don’t-care positions, *i.e.* if classical word-matches are counted, *d*_*RC*_ was slightly outperformed by *Co-phylog*, but was still better than all other methods in our comparison). The *RF* distance to the benchmark tree varied between 24 and 28. *Co-phylog* ranked second in this evaluation with a *RF* distance of 28. All other methods achieved a *RF* distance of greater or equal to 30. Surprisingly, *K*_*r*_ performs worse than other programs on these sequences.

### The variance of *N*: experimental results

**Figure 6:**
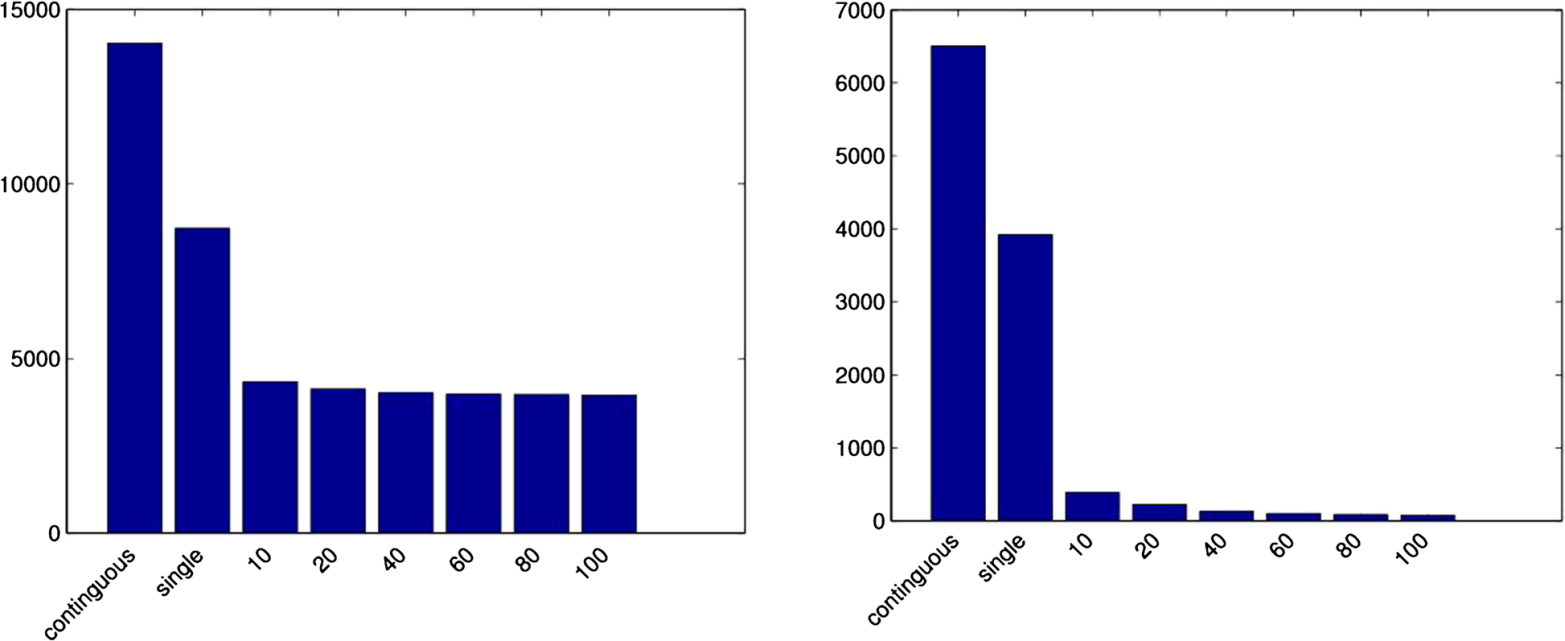
**Variance of the number of spaced-word matches.** Variance of the normalized number $\frac {N}{m}$ of spaced-word matches where $m={|\mathcal {P}|}$ is the number of patterns in the multiple-pattern approach. Formula (12) was applied to contiguous words and to single and multiple spaced words for un-gapped sequence pairs of length 16,000 *nt* with a mismatch frequency of 0.7 (left) and 0.25 (right).

Figure 1 shows not only striking differences in the shape of the distance functions used by various alignment-free programs. There are also remarkable differences in the *variance* of the distances calculated with our new distance measure *d*_*N*_ that we defined in equation (4). The distance *D*_*N*_ is defined in terms of the number *N* of *(spaced) word* matches between two sequences. As mentioned above, the established *Jensen-Shannon* and *Euclidean* distances on (spaced) word frequency vectors also depend on *N*, for small distances, they can be approximated by *L*−*N* and $\sqrt {L-N}$, respectively. Thus, the variances of these three distance measures directly depend on the variance of *N*. As can be seen in Figure 1, the variance of *d*_*N*_ increases with the frequency of substitutions. Also, the variance is higher for the single-pattern approach than for the multiple-pattern approach. To explain this observation, we calculated the *variance* of the normalized number *N*/*m* of spaced-word matches using equation 12. Figure 6 summarizes the results for a sequence length of *L*=16.000 and mismatch frequencies of 0.7 and 0.25, respectively. As can be seen, for single spaced words the variance of *N*/*m* is far smaller than for contiguous words, and for multiple spaced words, the variance is further reduced.

## Discussion

In this paper, we proposed a new estimator *d*_*N*_ for the evolutionary distance between two DNA sequences that is based on the number *N* of *spaced-word* matches between them. While most alignment-free methods use ad-hoc distance measures, the distance function that we defined is based on a probabilistic model of evolution and seems to be a good estimator for the number of substitutions per site that have occurred since two sequences have evolved separately. For simplicity, we used a model of evolution without insertions and deletions. Nevertheless, our test results show that our distance function is still a reasonable estimator if the input sequences contain a moderate number of insertions and deletions although, in this case, distances between the input sequences are overestimated since the number *N* of spaced-word matches is smaller than it would be for sequences without indels.

The model that we used to derive our distance *d*_*N*_ assumes that two sequences are globally related. If sequences share only local homology, the number *N* of spaced-word matches would be smaller than for globally related sequences with the same length and rate of mismatches, so their distance would be over-estimated by our distance measure *d*_*N*_. This is clearly a limitation of our approach. However, as indicated in section Estimating evolutionary distances from the number *N* of spaced-word matches, our distance function can be adapted to the case of *local* homologies if the length of these homologies and the number of gaps in the homologous regions can be estimated. In principle, it should therefore be possible to apply our method to locally related sequences by first estimating the extent of their shared (local) homology and then using the distance *d*_*loc*_ defined in equation (5) instead of *d*_*N*_.

The distance measures introduced in this paper and other distances that we previously used for our *spaced words* approach depend on the number *N* of space-word matches between two sequences with respect to a set  of patterns of ‘match’ and ‘don’t care’ positions. This is similar for more traditional alignment-free methods that calculated distances based on *k*-mer frequencies. While the *expected* number of (spaced) word matches is essentially the same for contiguous words and for spaced words of the corresponding *weight*, we have showed that the *variance* of *N* is considerably lower for spaced-words than for the traditionally used contiguous words. Moreover, with our *multiple-pattern* approach the variance of the normalized number of spaced-word matches is further reduced. This seems to be the main reason why our *multiple spaced words* approach outperforms the *single-pattern* approach that we previously introduced as well as the classical *k*-mer approach when used for phylogeny reconstruction.

As we have shown, the variance of *N* depends on the number of overlapping ‘match’ positions if patterns from  are shifted against each other. Consequently, in our single-pattern approach, the variance of *N* is higher for periodic patterns than for non-periodic patterns. For example, for the periodic pattern 101010…, the variance is equal to the variance of the contiguous pattern of the corresponding weight. In our previous benchmark studies, we could experimentally confirm that *spaced words* performs better with non-periodic patterns than with periodic patterns. The theoretical results of this study may be useful to find patterns or sets of patterns that minimize the variance of *N* to further improve our spaced-words approach.
